# Evaluation of Xanthine Oxidase Inhibitors Febuxostat and Allopurinol on Kidney Dysfunction and Histological Damage in Two-Kidney, One-Clip (2K1C) Rats

**DOI:** 10.1155/sci5/7932075

**Published:** 2025-01-22

**Authors:** Asif Ul Haque Shuvo, Mirza Alimullah, Ishrat Jahan, Kaniz Fatima Mitu, Md Junaeid Rahman, Kazi Akramuddaula, Ferdous Khan, Pritesh Ranjan Dash, Nusrat Subhan, Md Ashraful Alam

**Affiliations:** ^1^Department of Pharmaceutical Sciences, North South University, Dhaka, Bangladesh; ^2^Pharmacy Discipline, Khulna University, Khulna, Bangladesh; ^3^Department of Pharmacy, Primeasia University, Dhaka, Bangladesh

**Keywords:** allopurinol, febuxostat, inflammation, oxidative stress, uric acid

## Abstract

In chronic kidney disease (CKD), hyperuricemia is a common phenomenon, presumably due to reduced renal clearance of uric acid. This study investigated the effect of xanthine oxidase (XO) inhibitors allopurinol and febuxostat to prevent oxidative stress in the kidney of two-kidney, one-clip (2K1C) rats. In this investigation, 2K1C rats were used as an experimental animal model for kidney dysfunction. 2K1C rats were provided with food and drinking water and received febuxostat at a dose of 10 mg/kg or allopurinol at 100 mg/kg, respectively. After the treatment completion, all rats were sacrificed, and tissue samples were collected. 2K1C rats exhibited increased plasma creatinine, uric acid level, and glomerular injury assessed based on microscopic findings. Both allopurinol and febuxostat significantly normalized creatinine and uric acid levels. Furthermore, 2K1C rats showed increased lipid peroxidation (LPO), nitric oxide (NO), and advanced oxidation protein products (AOPP) alongside decreased superoxide dismutase (SOD) and catalase activity. Again, both drug treatments ameliorated these elevated oxidative stress parameters in 2K1C rats. The antioxidant genes such as Nrf-2, HO-1, and SOD were also restored in the kidneys of 2K1C rats by allopurinol and febuxostat treatment. 2K1C rats also showed increased IL-1β, IL-6, TNF-α, and NF-кB mRNA expression in the kidneys which were normalized by allopurinol and febuxostat treatment. Thus, the data suggest that XO inhibition protects kidney function potentially by restoring antioxidant enzyme function and suppressing inflammation.

## 1. Introduction

Chronic kidney diseases (CKDs) are increasing in developing countries like Bangladesh [1]. Major reasons for developing kidney dysfunction in Bangladesh are diabetes, hypertension, smoking habits, obesity salt intake in daily meals, etc. [2, 3]. The burden of kidney dysfunction in Bangladeshi patients is worsening due to the lack of dialysis facilities and tertiary care for CKD patients [4, 5]. Drug-induced kidney dysfunction is another real threat as increased trends have been found where drug-induced kidney dysfunction is growing due to NSAIDs and antiarthritic drugs [6]. Uric acid in urine is increased in renal insufficiency and is considered a marker of kidney dysfunction [7, 8]. Uric acid crystals have been demonstrated to possess the potential to bind to the surfaces of renal epithelial cells, triggering an immediate inflammatory response in the affected cellular lines [9]. Besides an elevated chance of renal calculi development, such impacts have also been demonstrated to diminish glomerular filtration rate (GFR) [10]. Subsequent investigations propose a correlation between heightened serum uric acid levels and the production of systemic cytokines, including tumor necrosis factor-α (TNF-α), along with the localized appearance of kidney chemokines, such as monocyte chemotactic protein 1 (MCP-1) [11, 12]. Increased cytokine production further aggravates the progression of chronic kidney failure [13, 14]. When it comes to lowering uric acid levels, xanthine oxidase (XO) inhibitors like febuxostat or allopurinol are indicated [10]. XO, an enzyme with a critical role, catalyzes the conversion of hypoxanthine to xanthine, transforms xanthine into uric acid, and stimulates the synthesis of reactive oxygen species (ROS). Allopurinol undergoes XO metabolism to produce oxypurinol; XO is inhibited by both substrates. Since the kidneys remove oxypurinol, the primary metabolite of allopurinol, patients with CKD may be more susceptible to toxicity from this medication. In patients with CKD, it is often advised to begin administering allopurinol at a low dose and gradually increase it to an effective dose [15]. This suggestion is also supported by the binding study of allopurinol and oxypurinol with XO advocated that readministration of allopurinol in constant small doses may be useful in inhibiting the XO [16]. Oxypurinol and allopurinol-induced inhibition of XO may increase the xanthine concentration in plasma and urine. Thus, allopurinol or oxypurinol-mediated toxicity may be due to increased xanthine levels, which are Stevens–Johnson syndrome and toxic epidermal necrolysis [17]. On the other hand, when allopurinol was administered to lower serum uric acid levels, both systolic and diastolic blood pressure significantly improved in comparison with a placebo [18]. Our previous study also suggests that allopurinol treatment may avert not only the oxidative stress but also the fibrosis in the heart of isoprenaline-administered rats [19]. It has been demonstrated that febuxostat is efficacious at lowering serum levels of uric acid as well. A recent meta-analysis showed that febuxostat treatment prevented kidney damage in CKD patients as well as lowered creatinine levels [20]. A postmarketing survey also favored the use of febuxostat over allopurinol about the manifestation of acute kidney [21]. However, the mechanism through which febuxostat restored renal function is not understood fully. A recent report suggests that febuxostat lowers blood pressure in hypertensive individuals [22]. Earlier reports postulated that febuxostat therapy may change the oxidative stress in hyperuricemia patients [23]. Oxidative stresses are a key regulator for the development of renal dysfunction [24]. Nrf-2 signaling, a master regulator for antioxidant enzymes in tissues, can be effective in protecting in contrast to renal failure in those with CKD [25]. This investigation will thus evaluate the oxidative stress along with related gene expression in the two-kidney, one-clip (2K1C) rat model as well as evaluate the therapeutic effect of febuxostat in kidney dysfunction in rats. Previous studies showed that 2K1C rats developed chronic kidney dysfunction by increasing the uric acid and creatinine levels [26]. Moreover, 2K1C animals indicated elevated oxidative stress and reduced antioxidant activities in plasma and tissues [26]. This project evaluated the febuxostat-induced alteration of kidney dysfunction in a suitable animal model. Moreover, this project also made a comparison between the allopurinol and febuxostat-mediated renal improvement in animal models.

## 2. Materials and Methods

### 2.1. Reagents and Chemicals

Uric acid (Ref. no. 30393), creatinine (Ref. no. 41262), and creatinine kinase-muscle/brain (CK-MB) (Ref. No. 41273) kits were also obtained from DCI Diagnostics (Budapest, Hungary). RevertAid First Strand cDNA Synthesis Kit (Catalog number: K1621), GeneJET RNA Purification Kit (Catalog number: K0732), and SYBR Green PCR Master Mix (Catalog number: 4309155) were purchased from Thermo Fisher Scientific Inc. (Waltham, Massachusetts, United State of America).

### 2.2. Animal and Experimental Design

Long Evans male rats (age 10–12 weeks, body weight 260–280 g) were used in this study. Twenty-four rats were taken from The Animal House of North South University, Bangladesh. Dark and light cycle (12 h) was maintained, and every rat was kept in separate cages (room temperature 25 ± 2°C, humidity 45%). Food and water were given freely to all rats, and all experimental procedures were approved by the IACUC of North South University, Bangladesh. The ethics approval number is 2020/OR-NSU/IACUC No. 1201.

### 2.3. 2K1C Surgical Procedure

The surgical procedure was performed to develop the 2K1C rats where a kidney artery was clipped with a plastic tube (2 mm long and 0.4 mm internal diameter) using the butterfly needle tubing as described previously [27]. The animals were anesthetized with 50 mg/kg ketamine (i.p.). The skin was shaved carefully. A small incision was made on the left side beside the spine. The left kidney was carefully taken out from the peritoneal cavity, and the adjacent adipose tissues were cleared with a sterile tweezer. For clipping, the renal artery and ureter of the left kidney were separated clearly [27]. The plastic clip was placed around the renal artery carefully and tightened with the help of a suture knot. This clipping resulted in the partial occlusion of renal perfusion. The kidney was then gently pushed back into the retroperitoneal cavity. The blood was cleared with povidone-iodine, and the incision was closed layer by layer with sutures. Lidocaine gel was applied to the wound. A sham surgical procedure was also performed without clipping the artery. At the end of the experiment, the left kidney shirked significantly compared to the right kidney [28]. The animals were kept in closed monitoring for full recovery and placed in the experimental groups.

### 2.4. Animals' Groups and Treatment

The following groups were developed to study the effect of allopurinol and febuxostat in 2K1C rats:

a. Group I (control, *n* = 6). These rats received only the control diet and received the shame surgery without clipping.
b. Group II (2K1C, *n* = 6). These rats experienced 2K1C surgery procedures and were later supplied control diet and water.
c. Group III (2K1C + Febuxostat, *n* = 6). These rats experienced 2K1C surgery procedures and were later received 10 mg/kg febuxostat.
d. Group IV (2K1C + Allopurinol, *n* = 6). These rats experienced 2K1C surgery procedures and were later received 100 mg/kg allopurinol.

The animals were treated with allopurinol and febuxostat for 4 weeks, and after that, all animals were sacrificed. The animal sacrifice was done using a high dose of pentobarbital aesthesis (90 mg/kg). The blood samples were collected from the abdominal vein using an 18-gauge needle and syringe and placed in citrate buffer solution (pH 4.2) containing tube for the preservation in the freezer (−20°C) for future analysis. The internal organs such as heart and kidneys were also collected and weighed immediately after the sacrifice. One part of the tissues was preserved in neutral buffered formalin solution (pH 7.4), and the other parts were placed in cryopreservation tube and placed in −80°C freezer. The preserved samples were used in the next biochemical analysis. A separate part from kidney cortex was also preserved for mRNA extraction process and RT-PCR process. Expression levels of several stress signaling molecules like Nrf-2, HO-1, NF-кB, and superoxide dismutase (SOD) were assessed through PCR using isolated kidney mRNA samples. Plasma biochemical analysis was conducted for oxidative stress markers, cardiac, and kidney-specific markers. Histological staining was performed on formalin-fixed tissues after slicing, with hematoxylin–eosin (H&E) stain and Sirius red stain in the heart and kidneys.

### 2.5. Preparation of Tissue Sample for the Assessment of Oxidative Stress Markers

Collection of heart and kidney samples was carried out for the purpose of evaluating oxidative stress markers and blended into a homogeneous mixture using a pH 7.4 phosphate buffer at a 10:1 buffer volume to tissue ratio. Following the initial preparations, the suspensions were subjected to high-speed centrifugation at 5000 × g for 30 min at 4°C, leading to the distinct separation of soluble supernatants and insoluble pellets. After centrifugation, the supernatants were retained and subsequently utilized in further experimental investigations.

### 2.6. Lipid Peroxidation (LPO) Assay as Malondialdehyde (MDA)

Using the previously reported procedure, quantitative assessment of thiobarbituric acid reactive substances (TBARS) was achieved colorimetrically to quantify LPO in the heart along with kidney [29]. In the simplest terms, a mixture of 2 mL of TBA-TCA-HCl reagent (with thiobarbituric acid 0.37%, 0.25 N HCl, and 15% TCA in a 1:1:1 ratio) was combined with 0.1 mL of tissue homogenate (Tris-HCl buffer, pH 7.5). The resulting mixture underwent a 15-min heat treatment in a hot water bath and was subsequently cooled to room temperature. Afterward, the clear supernatant's absorbance at 535 nm was measured, and this value was compared to a reference blank.

### 2.7. Nitric Oxide (NO) Assay

NO was deduced in keeping with the process outlined by Tracy et al. as nitrate and nitrite [30]. Instead of utilizing 1-naphthylamine (5%), N-(1-naphthyl)ethylenediamine dihydrochloride (0.1% w/v) was used in this investigation to modify the Griess-Ilosvay reagent. Incubation at 25°C for 150 min was carried out for the reaction mixture (3 mL), which included tissue homogenates or plasma sample (2 mL) along with phosphate buffer saline (0.5 mL). These solutions were subjected to absorbance measurement at 540 nm, and comparison was made to the correlative blank solutions. Using a standard curve, the NO level was measured and reported as nmol/mL. Utilizing a standard curve, the measurement of the NO level was conducted, and the reported values were expressed in nmol/mL.

### 2.8. Advanced Oxidation Protein Products (AOPP) Assay

Determination of AOPP amounts was conducted through adjustment of the technique of Witko-Sarsat et al. [31] and Tiwari et al. [32]. Following the addition of 0.1 mL of 1.16 M potassium iodide to each tube, 0.2 mL of acetic acid was introduced after a two-minute interval. The dilution process will result in a 1:5 ratio in PBS for 2 mL of plasma. Following that, the reaction mixture's absorbance was measured at 340 nm in correspondence with a blank control that included 0.2 mL of acetic acid, 0.1 mL of KI, and 2 mL of phosphate buffer saline (PBS, pH 7.4).

### 2.9. Catalase (CAT) Activity Assay

CAT activities were determined by following the Khan technique delineated previously adding a few adjustments [33]. The assay to quantify CAT activities involved a reaction solution containing the following: 2.5 mL of 50 mmol pH 5.0 phosphate buffer, 0.4 mL of 5.9 mmol H_2_O_2_ solution, and 0.1 mL of enzyme extract; monitoring absorbance changes at 240 nm 1 min postinitiation.

### 2.10. Estimation of SOD Activity

In the case of plasma and tissue homogenates, SOD assay was performed according to a previously described method [34]. PBS was added with 3 mL of the reaction mixture which consists of an aliquot of enzyme preparation making volume up to 2.94 mL. 0.06 mL adrenaline (15 mM) was added to start the reaction. At 15-s interval for 1 min, the absorbances were recorded at 490 nm.

### 2.11. Reduced Glutathione (GSH) Assay

Measurement of reduced GSH utilized documented methodology formerly outlined by Jollow et al. [30]. 10% tissue homogenate (1 mL) was precipitated using equal volume of 4% sulfosalicylic acid (1 mL) for 1 h at 4°C and centrifuged, and the GSH assay was performed by mixing 0.1-mL filtered aliquot with buffer (2.7 mL of 0.1 M pH7.4 phosphate) and 0.2 mL DTNB reagent (100 mM 5,5-dithiobis-2-nitrobenzoic acid) measuring absorption at 412 nm instantly to determine reduced GSH content normalized by protein concentration. Results were expressed per mg protein.

### 2.12. Estimation of Myeloperoxidase (MPO) Activity

The dianisidine-H_2_O_2_ system-based method was used to determine MPO activities in kidney homogenates modified for 96-well plates [35]. In the sample mixture, the contents were as such-kidney sample was about 10 μg protein, o-dianisidine dihydrochloride (0.53 mM), and hydrogen peroxide (0.15 mM) in potassium phosphate buffer (50 mM) (pH 6.0). Absorbance was taken at 450 nm. Units were expressed as MPO/mg protein.

### 2.13. Uric Acid, Creatinine, and CK-MB Assay

Uric acid and creatinine concentrations in the plasma were estimated using the appropriate kits provided by DCI Diagnostics (Budapest, Hungary) following the manufacturer's supplied protocol in a semiautomatic analyzer (Clindiag, Zhenjiang, China). The CK-MB activity in plasma was also determined by a kit collected from DCI Diagnostics (Budapest, Hungary) following the manufacturer's supplied protocol.

### 2.14. RT-PCR for Oxidative Stress and Inflammation Regulatory Genes

Total mRNA was isolated and purified from the kidney of all four rat groups using the GeneJET RNA Purification Kit provided by Thermo Fisher Scientific (Massachusetts, USA) after the sacrifice for blood and organ collection. Accurate measurement and reliable data analysis of mRNA concentration were ensured through the utilization of the NanoDrop 2000 spectrophotometer by Bio-Rad (California, USA). The cDNA was synthesized using Thermo Fisher Scientific RevertAid First Strand cDNA Synthesis Kit, which is based in the USA. 1 μg of mRNA from each sample was used in this procedure, which was carried out in a T100 Thermal Cycler manufactured by California-based Bio-Rad following concentration measurements. Using Maxima SYBR Green qPCR master mixes (Thermo Scientific, USA) and a CFX96 C1000 Touch Real-Time PCR Detection System (Bio-Rad, California, USA), the mRNA levels of transcription factors and enzymes associated with oxidative stress and inflammation were measured from the extracted cDNA. The manufacturer's instructions for the CFX Manager Software (CFX Manager Software) were followed after the data analysis. The primers indicated in Table 1 are oligonucleotides [36] that were built using the online Primer 3 program and will be utilized as both forward and reverse primers in the quantitative real-time PCR. Amplification of target genes was conducted through a one-minute polymerase chain reaction at 95°C, with 40 cycles comprising denaturation at 95°C for 5 s, annealing at 60°C for 30 s, extension at 72°C for one minute, and final extension at 72°C for five minutes. The transcript levels of each gene were determined by normalization to the β-actin control.

### 2.15. Histopathological Studies

The heart tissues underwent a series of graded ethanol and xylene treatments after a fixation in 10% neutral buffered formalin. Next, the tissues were immersed in paraffin blocks, while a rotary microtome was used to cut sections of the tissues that were roughly 5 μm thick. H&E will be treated to these sections in accordance with standard protocol. The heart and kidney slices were additionally stained with Sirius red staining for fibrosis. After that, sections were examined and captured on camera at 40X magnification using a light microscope. Pathomorphological alterations on the slides were investigated.

The EGTI scoring system-based histological scoring system was used to measure kidney damage in laboratory animals, in which *endothelial, glomerular, tubular*, and *interstitial* damages are considered and were examined [37, 38]. Estimation of fibrosis percentages in kidney and cardiac areas involved the use of the ImageJ free software (Version 4.0) provided by the National Institute of Health in the United States of America [36, 38].

### 2.16. Molecular Docking

#### 2.16.1. Protein/Receptor Preparation

The protein/receptor, human milk xanthine oxidoreductase/XO (PDB ID: 2CKJ) as Protein Data Bank, provided the PDB format for downloading (https://www.rcsb.org/). By using PyMOL, water molecules and original ligands were deleted. Autodock tools 1.5.7 was used to prepare the protein, and polar hydrogen and Kollamn charge were added to the prepared protein. The receptor/protein was saved as PDBQT format [39–41].

#### 2.16.2. Ligand Preparation

The 3D structure of ligand allopurinol (PubChem ID: 135401907) and the SDF format of febuxostat (PubChem ID: 134018) have been obtained from PubChem (https://pubchem.ncbi.nlm.nih.gov/). PDB format is required for using AutoDock Tools, and therefore, using PyMOL, SDF files were converted into PDB format. By using AutoDock Tools 1.5.7, the ligands were prepared for docking and saved as PDBQT file [40, 41].

#### 2.16.3. Grid Preparation

AutoGrid parameters were computed using the grid menu available in AutoDock Tools (10). The protein was uploaded as PDBQT format, and from the grid menu, grid box was selected, and a box appeared. The Center *X* = 4.553, *Y* = 77.579, and *Z* = 118.879, and the default dimensions of *X*, *Y*, *X* coordinates were 40 × 40 × 40, respectively. The grid file was saved as GPF format [40, 41].

#### 2.16.4. Docking

AutoDockVina is a complete computational docking method based on a quick conformational search and a basic scoring system [42]. The default techniques in AutoDock and AutoDockVina have been extensively utilized for applications like virtual screening since they are quite efficient for typical drug-like ligands [43]. AutoDockVina was run by using command prompt [42], and the docked file was saved as PDBQT format [41].

#### 2.16.5. Visualization

The 2D and 3D structures of docked protein and ligand were visualized by using Bovia Discovery Studio Client 2021 [44].

### 2.17. Data Analysis

Statistical analysis was done by Prism software for Windows operating system. Data are being presented as mean ± SEM. These results are analyzed by one-way analysis of variance followed by the Tukey test to determine differences between treatment groups; *p* < 0.05 is considered as significant. Additionally, data obtained from the kidney tissue biochemical parameters were subjected to principal component analysis (PCA) using PAST software, version 4.03.

## 3. Results

### 3.1. Effect of Allopurinol and Febuxostat on Kidney and Heart Wet Weight of 2K1C Rats

**Figure 1:**
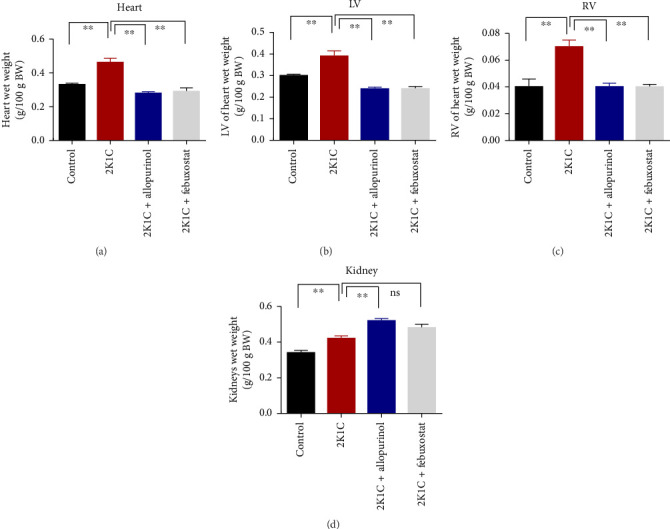
Effect of allopurinol and febuxostat on kidney and heart wet weight of 2K1C rats. Data are expressed as mean ± SEM, *n* = 6. Statistical analysis was done by one-way ANOVA followed by Tukey's post hoc test. Statistical significance was considered as *p* < 0.05.

Effect of allopurinol and febuxostat on kidney and heart wet weight of 2K1C rats is expressed in Figure 1. The wet weights of heart, LV, and RV were increased significantly (*p* < 0.05) in 2K1C rats compared to control rats. The wet weights of heart, LV, and RV were decreased significantly (*p* < 0.05) with the treatment of allopurinol and febuxostat in 2K1C rats (Figure 1). The kidney wet weight of the rats in 2K1C rats was also increased significantly (*p* < 0.05) compared to the control group. When compared to 2K1C rats, the kidney wet weight of rats treated with allopurinol was significantly higher, while rats treated with febuxostat did not exhibit a significant change in kidney wet weight (Figure 1).

### 3.2. Effect of Febuxostat and Allopurinol on Oxidative Stress Markers (MDA and NO) and Antioxidant Enzymes

**Figure 2:**
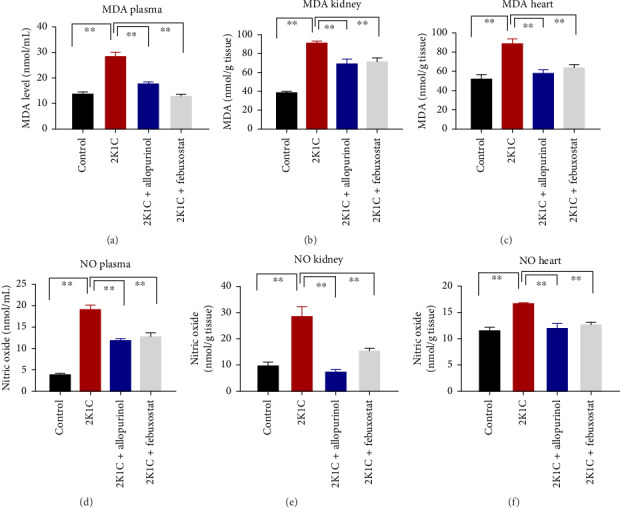
Effect of allopurinol and febuxostat on oxidative stress parameters such as MDA and NO in plasma, kidney, and heart tissue homogenates of 2K1C rats. Data are expressed as mean ± SEM, *n* = 6. Statistical analysis was done by one-way ANOVA followed by Tukey's post hoc test. Statistical significance was considered as *p* < 0.05 and marked as an asterisk mark. APOP = advanced protein oxidation product; MDA = malondialdehyde; NO = nitric oxide.

To determine the oxidative stress, we assayed the oxidative stress parameters such as MDA and NO in the plasma, kidney, and heart tissues, respectively (Figure 2). In this investigation, it was found that the levels of MDA were significantly (*p* <  0.05) elevated in plasma, kidney, and heart of 2K1C rats. To add on, in comparison with 2K1C group, febuxostat, and allopurinol treatment reduced the levels of LPO products MDA in the plasma, kidney, and heart tissue homogenates in the plasma, kidney, and heart tissue homogenates (Figure 2).

Furthermore, significant increases (*p* < 0.05) in NO levels, measured as nitrate/nitrite, were detected in the plasma and kidney tissue of 2K1C rats compared to both the control group and control + febuxostat rats (Figure 2). Administration of febuxostat and allopurinol considerably decreased the nitrate/nitrite levels in plasma, kidney, and heart of 2K1C rats (Figure 2).

### 3.3. Effect of Febuxostat and Allopurinol on AOPP, CK-MB, and MPO Activity

**Figure 3:**
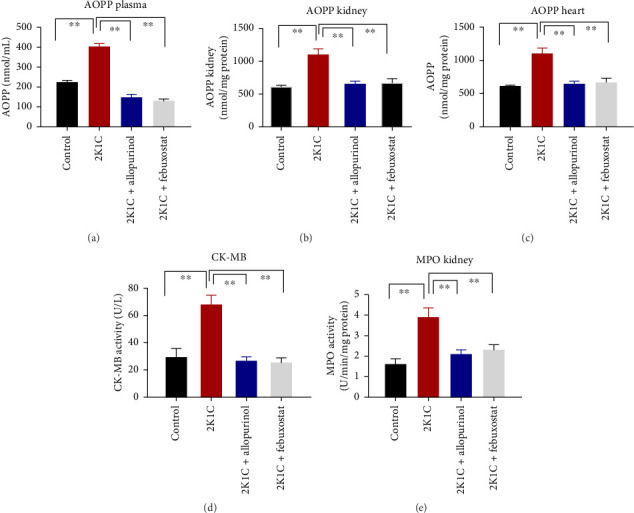
Effect of allopurinol and febuxostat on oxidative stress parameters such AOPP in plasma, kidney, and heart tissue homogenates as well as CK-MB activities in plasma of 2K1C rats. Data are expressed as mean ± SEM, *n* = 6. Statistical analysis was done by one-way ANOVA followed by Tukey's post hoc test. Statistical significance was considered as *p* < 0.05 and marked as an asterisk mark. APOP = advanced protein oxidation product.

Another essential indicator of oxidative stress in tissues is the AOPP which is obtained as a product from the interaction between chlorine free radicals with plasma proteins. As per Figure 3, the 2K1C rats showed substantially high AOPP concentrations in plasma, kidney, and heart tissues (*p* < 0.05), which was brought down to normal levels by febuxostat and allopurinol treatment (Figure 3). Similarly, the plasma levels of CK-MB in 2K1C rats were also increased significantly (*p* < 0.05) compared to the control (Figure 3). Addition of febuxostat and allopurinol in the 2K1C rats decreased CK-MB activities significantly compared to 2KC rats (Figure 3).

MPO is a sign of inflammatory cells as it is expressed mostly in the phagocytes. The MPO activities were significantly (*p* < 0.05) elevated in the kidney homogenates of 2K1C rats compared to the control (Figure 3). Treatment with febuxostat and allopurinol in the 2K1C rats lowered the MPO activities significantly (*p* < 0.05) compared to the 2KC rats (Figure 3).

### 3.4. Effect of Febuxostat and Allopurinol on Plasma Creatinine and Uric Acid Level

**Figure 4:**
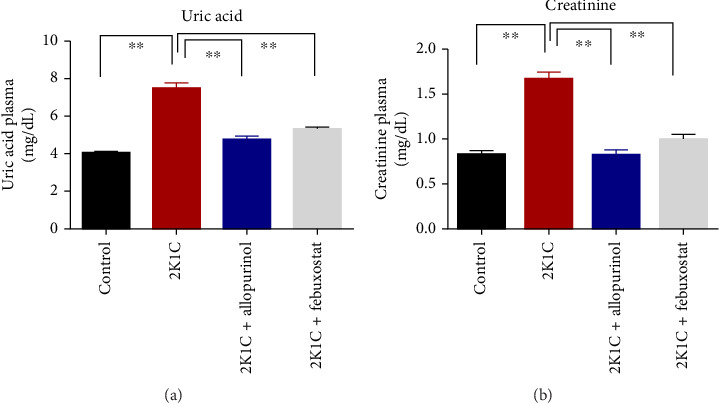
Effect of allopurinol and febuxostat on uric acid and creatinine in plasma of 2K1C rats. Data are expressed as mean ± SEM, *n* = 6. Statistical analysis was done by one-way ANOVA followed by Tukey's post hoc test. Statistical significance was considered as *p* < 0.05 and marked as an asterisk mark.

The effect of allopurinol and febuxostat on uric acid and creatinine is presented in Figure 4. The levels of creatinine and uric acid were significantly elevated (*p* < 0.05) in 2K1C rats relative to control rats (Figure 4). The significant reduction in plasma uric acid and creatinine levels in 2K1C rats treated with allopurinol and febuxostat indicated a noteworthy improvement in kidney function compared to the 2K1C rats (Figure 4).

### 3.5. Effect of Febuxostat and Allopurinol on Antioxidant Enzymes Like SOD and CAT Activities as Well as GSH Levels in Plasma, Kidney, and Heart of 2K1C Rats

**Figure 5:**
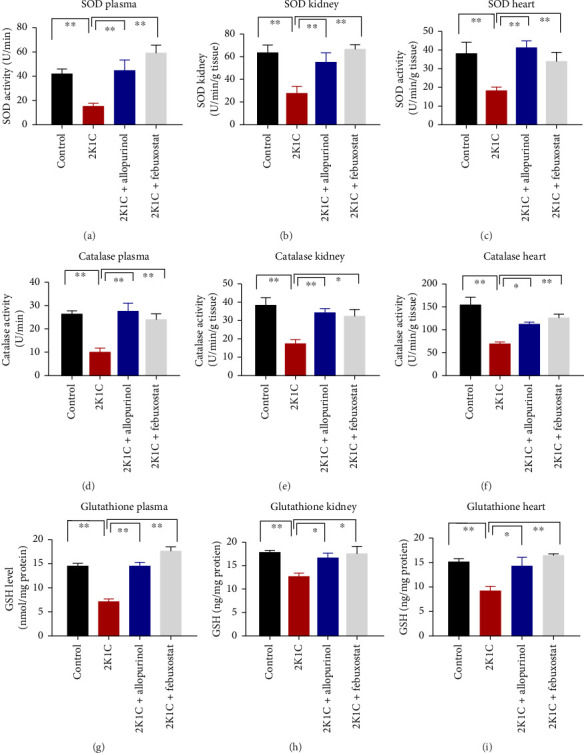
Effect of allopurinol and febuxostat on antioxidant enzyme activities such as SOD and catalase activities and glutathione level in plasma, kidney, and heart tissue homogenates of 2K1C rats. Data are expressed as mean ± SEM, *n* = 6. Statistical analysis was done by one-way ANOVA followed by Tukey's post hoc test. Statistical significance was considered as *p* < 0.05 and marked as an asterisk mark.

Antioxidant enzymes like SOD and CAT activities as well as GSH levels in plasma, kidney, and heart tissues are presented in Figure 5. In 2K1C rats, the activity of SOD in plasma, kidney, and heart displayed a noteworthy decrease (*p* < 0.05) in comparison with the control group (Figure 5). On treatment with allopurinol and febuxostat, the SOD activity restored significantly in 2K1C rats (Figure 5).

In 2K1C rats, there was a significant decline (*p* < 0.05) in CAT activity in plasma, kidney, and heart tissues compared to the control group (Figure 5). Administration of allopurinol and febuxostat both restored the CAT activity in plasma, kidney, and heart tissues of 2K1C rats (Figure 5).

The GSH level in plasma, kidney, and heart showed a significant decrease (*p* < 0.05) in 2K1C rats relative to the control group (Figure 5). Treatment with allopurinol and febuxostat individually restored the GSH levels back to normal in all the tissues of 2K1C rats (Figure 5).

### 3.6. PCA for the Biochemical Parameters From the Kidneys of 2K1C Rats

**Figure 6:**
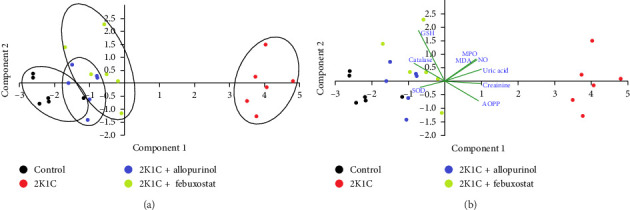
Principal component analysis (PCA) biplot graph of biochemical parameters of the kidney in various groups. AOPP, advanced oxidation protein product; GSH, reduced glutathione; MDA, malondialdehyde; MPO, myeloperoxidase; NO, nitric oxide; SOD, superoxide dismutase. Blue fonts and green lines show correlations of measured parameters with regard to experimental groups in the PCA plot (autoscaled). The PCA from the kidney showed a prominent separation of groups across Component 1, accounting for more than 67.14% of observed variation. The control group (black dots) separated sharply to the left, whereas the 2K1C (brown dots) clustered on the right-hand side of the plot. This separation was statistically significant (*p*  <  0.001). The 2K1C + allopurinol (blue dots) and 2K1C + febuxostat (green dots) made clustering between other groups mostly on the left side of the plot with regard to both the Component 1 and Component 2 axes. The main variables contributing to the separation across Component 1 were AOPP, NO, uric acid, and creatinine, correlating with the 2K1C group on the right-hand side, whereas the GSH level and the catalase activity correlated with the control group on the left-hand side of the plot.

The PCA from the kidney showed a prominent separation of groups across Component 1, accounting for more than 67.14% of observed variation. The control group (black dots) separated sharply to the left, whereas the 2K1C (brown dots) clustered on the right-hand side of the plot. This separation was statistically significant (*p* < 0.001). The 2K1C + allopurinol (blue dots) and 2K1C + febuxostat (green dots) made clustering between other groups mostly on the left side of the plot with regard to both the Component 1 and Component 2 axes. The main variables contributing to the separation across Component 1 were AOPP, NO, uric acid, and creatinine, correlating with the 2K1C group on the right-hand side, whereas the GSH level and the CAT activity correlated with the control group, on the left-hand side of the plot (Figure 6).

### 3.7. Effect of Allopurinol and Febuxostat on Antioxidant Enzyme Gene Expression in the Kidney Cortex of 2K1C Rats

**Figure 7:**
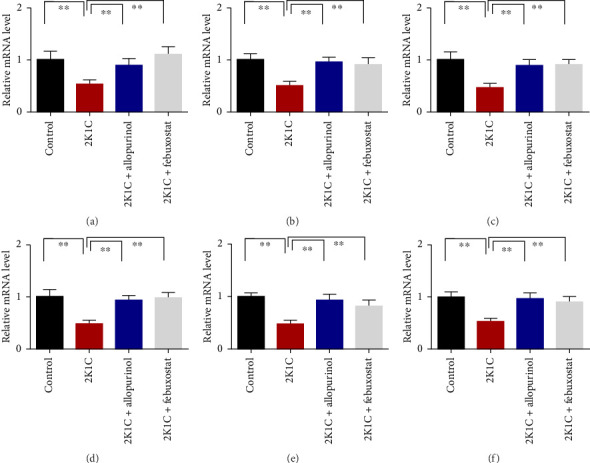
Effect of allopurinol and febuxostat on antioxidant enzyme gene expression in the kidney cortex of 2K1C rats. Data are expressed as mean ± SEM, *n* = 6. Statistical analysis was done by one-way ANOVA followed by Tukey's post hoc test. Statistical significance was considered as *p* < 0.05 and marked as an asterisk mark. (a) NRF2, (b) HO-1, (c) HO-2, (d) SOD, (e) GPx, and (f) catalase.

Figure 7 shows the effect of allopurinol and febuxostat on antioxidant enzyme gene expression in the kidney cortex of 2K1C rats. The relative mRNA expression of Nrf-2 declined significantly (*p* < 0.05) for 2K1C rats compared to control rats (Figure 7). The administration of allopurinol and febuxostat on individual basis showed significant restoration of mRNA expression for Nrf-2 (Figure 7). Nrf-2 mediated other genes such as HO-1 and HO-2 gene expressions are also decreased significantly (*p* < 0.05) in the kidney cortex of 2K1C rats (Figure 7). Allopurinol and febuxostat treatment restored the HO-1 and HO-2 gene expressions in 2K1C rats (Figure 7). Furthermore, Nrf-2-mediated decline could result in the decreased SOD, GPx, and CAT gene expressions in the kidney of 2K1C rats, which was further improved by the treatment with allopurinol and febuxostat (Figure 7).

### 3.8. Effect of Allopurinol and Febuxostat on Inflammation-Related Gene Expression in the Kidney Cortex of 2K1C Rats

**Figure 8:**
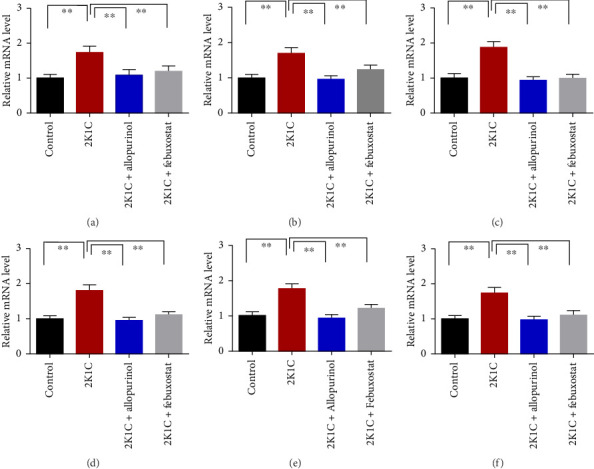
Effect of allopurinol and febuxostat on inflammation-related gene expression in the kidney cortex of 2K1C rats. Data are expressed as mean ± SEM, *n* = 6. Statistical analysis was done by one-way ANOVA followed by Tukey's post hoc test. Statistical significance was considered as *p* < 0.05 and marked as asterisk mark. (a) IL-1, (b) IL-6, (c) TNFα, (d) TGFβ, (e) iNOS, and (f) NF-кB.

Figure 8 enumerates the effect of allopurinol and febuxostat on inflammation-related gene expression in the kidney cortex of 2K1C rats. These results show that the relative mRNA expression for inflammatory genes such as IL-1, IL-6, and TNF-α was increased almost doubled (*p* < 0.05) for 2K1C rats compared to control rats (Figure 8). Administration of allopurinol and febuxostat prevented the rise of these inflammatory gene expressions in the kidney cortex of 2K1C rats significantly to the same level as of control rats (Figure 8).

The increased inflammatory gene expressions could be attributed to the increased expression of NF-κB in the kidney cortex of 2K1C rats (Figure 8), which was normalized by the treatment with allopurinol and febuxostat. 2K1C rats also showed increased fibrogenic gene expression TGF-β in kidney cortex significantly compared to the control rats (Figure 8). Allopurinol and febuxostat prevented the expression TGF-β in kidney cortex in 2K1C rats. Moreover, the expression of iNOS in the kidney cortex significantly increased in 2K1C rats relative to the control rats (Figure 8). Allopurinol and febuxostat prevented the expression iNOS in kidney cortex in 2K1C rats (Figure 8), which may be a reason of decreased NO level in the kidney tissues (Figure 8).

### 3.9. Effect of Allopurinol and Febuxostat on Histomorphology in the Remnant Kidney of 2K1C Rats

**Figure 9:**
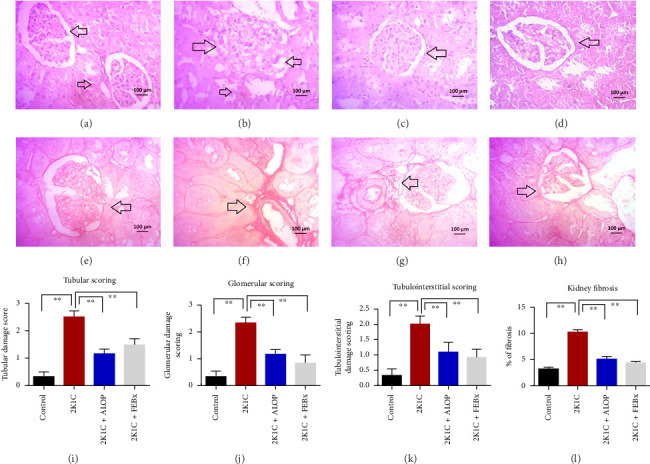
Effect of allopurinol and febuxostat on histological structure in the kidney of 2K1C rats. Upper panel—H and E staining. Lower panel—Sirius red staining. (a) Control showed normal histoarchitecture of the glomerulus and proximal and distal tubular linings with no damage and necrosis in the kidney. (b) 2K1C, enlarged glomerulus and disoriented glomerular orientation were observed. The proximal and distal tubular linings were broken and became thick compared to the control rats. (c) 2K1C + allopurinol showed improved glomerular orientation and improved proximal and distal tubular lining compared to the 2K1C. (d) 2K1C + febuxostat also showed improved glomerular orientation compared to the 2K1C. These observations are supported by the EGTA scoring system showed that tubular, glomerular, and tubulointerstitial damages were observed in 2K1C rats compared to the control (i–k) which were reduced by allopurinol and febuxostat treatment. (e) Control, Sirius red staining showed normal collagen baseline in the tissue section. (f) 2K1C showed increased collagen deposition and fibrosis around the blood vessels and tubules in the kidney. (g) 2K1C + allopurinol showed decreased collagen deposition and fibrosis in the kidney section compared to the 2K1C. (h) 2K1C + febuxostat showed decreased collagen deposition and fibrosis in the kidney section compared to the 2K1C. The % of fibrosis presented in the graph L showed increased % of fibrosis in the 2K1C kidneys compared to control rats which was reduced by allopurinol and febuxostat treatment. Data are expressed as mean ± SEM, *n* = 3. Statistical analysis was done by one-way ANOVA followed by Tukey's post hoc test. Statistical significance was considered as *p* < 0.05 and marked as an asterisk mark.

Renal artery clipping caused the clipped kidney to gradually atrophy, while the nonclipped kidney showed compensatory hypertrophy. Consequently, an evaluation of the experimental groups' renal anatomy and function was conducted.  Figure 9 depicts the nonclipped kidney structures. Four weeks after clipping, histological staining was not carried out as the clipped kidney progressed into renal atrophy in both the 2K1C and 2K1C + treatment groups.

After 4 weeks of clipping, the renal histology of the unclipped kidney showed that the 2K1C group had more glomerulosclerosis and interstitial fibrosis than the control group (Figure 9). The 2K1C rats' glomerulosclerosis and interstitial fibrosis were considerably improved by allopurinol and febuxostat therapy (Figure 9). The EGTI scoring system was also used to assess *tubular*, *glomerular,* and *interstitial* damage in kidney sections. *Tubular*, *glomerular,* and *interstitial* damages are found significantly increased in 2K1C rats compared to the control rats (Figure 9). Allopurinol and febuxostat treatment showed significant (*p* < 0.05) lowering of *tubular*, *glomerular,* and *interstitial* damages in the kidney of the 2K1C rats (Figure 9).

**Figure 10:**
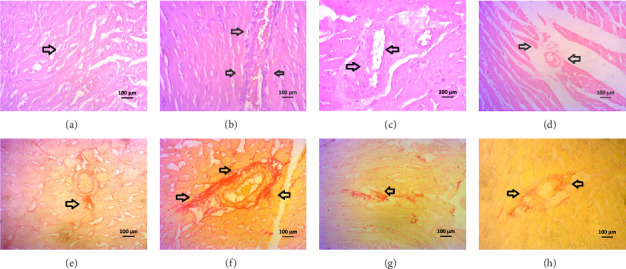
Effect of allopurinol and febuxostat on the histological structure in the heart of 2K1C rats. Upper panel—H and E staining. Lower panel—Sirius red staining. (a) Control, (b) 2K1C, (c) 2K1C + allopurinol, and (d) 2K1C + febuxostat. Lower panel shows Sirius red staining for collagen fibers, (e) control, (f) 2K1C, (g) 2K1C + allopurinol, and (h) 2K1C + febuxostat. Magnifications 40x.

The H&E staining of the left ventricle of the heart showed normal architecture in control rats, while inflammatory cells are seen in the heart of 2K1C rats (Figure 10, upper panel). Allopurinol and febuxostat treatment in 2K1C rats lowered the inflammatory cells in the heart (Figure 10, upper panel). The Sirius red staining in the heart also showed no fibrosis and collagen deposition in control rats which was increased in 2K1C rats (Figure 10, lower panel). Allopurinol and febuxostat treatment lowered the fibrosis in the heart of 2K1C rat (Figure 10, lower panel).

### 3.10. Computational Study

**Figure 11:**
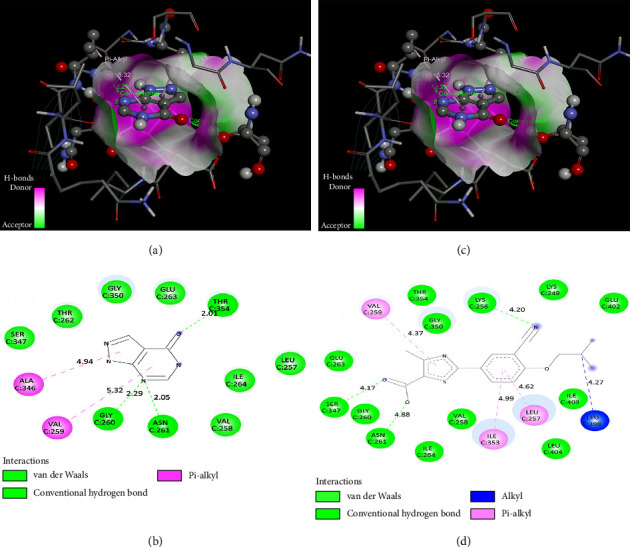
Best rank pose of interaction of allopurinol and xanthine oxidase 3D (a) and 2D (b), and best rank pose of interaction of febuxostat and xanthine oxidase 3D (c) and 2D (d).

Molecular docking was done between human milk xanthine oxidoreductase/XO (PDB ID: 2CKJ), and allopurinol and febuxostat. Visual interaction of XO (PDB ID: 2CKJ) with allopurinol and febuxostat is shown in Figure 11, and their binding information is given in Table 2.

## 4. Discussion

This study pinned down that the treatment with the XO inhibitors allopurinol and febuxostat reduced oxidative stress, inflammatory cell infiltration, and fibrosis in the kidney of 2K1C rats. As per the previous research, the renin–angiotensin–aldosterone axis is continuously activated in the 2K1C rat model, due to the kidney's overproduction of renin and lower renal perfusion pressure [45]. This situation may occur in humans as well due to renal artery stenosis, reasonably caused by atherosclerotic or fibromuscular dysplastic renal disease [46]. The result of the kidney damage brought about by 2K1C surgery, oxidative stress, and fibrosis eventually develop, and because of this pathophysiological condition, the 2K1C model has been utilized as a reliable animal model for studying renal disease for decades [47].

The 2K1C rats showed an increased weight in nonclipped kidney compared to the control rats. The nonclipped kidney weights were also increased in allopurinol and febuxostat-treated rats. These changes in the nonclipped kidney weight could be compensatory mechanism due to the damage and nonfunctional clipped kidney. This finding is supported by a previous report showing that 2K1C rats showed increased nonclipped kidney weights compared to the sham rats [28]. The increase in nonclipped kidney weights and hypertrophy relies on the presence of glomerular hyperperfusion, hyperfiltration, and glomerular hypertension [48, 49]. The hypertension and increased renin level are also implicating the hypertrophy signal in 2K1C rats [50]. However, the limitations of this study were the unavailability of blood pressure data and renin level in plasma. Considering other references, this kidney hypertrophy in this investigation may depend on the renin–angiotensin-induced oxidative stress in the nonclipped kidneys. In this study, the allopurinol treatment further increased the kidney wet weight compared to 2K1C rats, which was not observed in febuxostat treatment. The allopurinol and febuxostat both target similar pathways in purine metabolism and inhibit the XO enzyme. A lack of literature was found on the effect of allopurinol in changing the cellular growth or hypertrophy. An overall health improvement due to allopurinol treatment could be a reason for the increased kidney wet weight in 2K1C rats.

A proposed theory indicates that oxidative stress notably contributes to the molecular changes underlying the experimental kidney injury in these animal models. The rise in the production of reactive oxygen species (ROS) resulting from a diminished blood flow to one kidney after 2K1C surgery activates the renin–angiotensin system and eventually propagates to the development of oxidative stress in the renal system [51]. The excess ROS sequentially oxidize cellular macromolecules followed by the oxidation of membrane phospholipids. The prime targets of such ROS-induced oxidation are cardiomyocytes and blood vessels. Attacking the lipid layers of the cell membrane, these free radicals produced in tissues can destroy the cell membrane, causing cellular necrosis and tissue damage [52]. The oxidation of membrane lipids can be measured as MDA. The raised production of free radicals in tissues is the outcome of a number of causes, including aberrant mitochondrial function, NADPH and iNOS activity, chemical mediators, and industrial pollutants [53, 54]. Angiotensin II is a naturally occurring mediator that may control cellular ROS generation, mitochondrial activity, and NADPH activities [55, 56]. According to a previous study, 2K1C rats have overactivated Angiotensin II pathway and went to develop hypertension and endothelial impairment due to increased free radicle-mediated oxidative stress in 2K1C rats [57]. In animal models, antioxidants have a protective effect against renal and cardiac dysfunction brought about by reducing free radical production and by the prevention of LPO and MDA formation. The findings of this study imply that allopurinol and febuxostat have antioxidant and protective effects against renal and cardiac dysfunction in 2K1C rats by decreasing the MDA formation. An earlier study has shown that allopurinol and febuxostat treatment protects against renal failure and reduces oxidative stress in the kidneys [58, 59]. In fact, XO, in its biochemical pathway in the conversion of purine derivatives to uric acid, produces the superoxide anion [^−^O^•^_2_] and hydrogen peroxide (H_2_O_2_) [60]. Thus, inhibition of XO by allopurinol and febuxostat may decrease the free radicle production and oxidative stress [61]. This investigation also revealed that 2K1C rats showed elevated AOPP levels. Protein product is oxidized due to the reaction of free radicles from hypochlorous acid origin and mostly related to the MPO activities [62]. Previous report suggests that AOPP is responsible for the development of kidney complications in both humans [63, 64] and experimental animals [19]. Previously, we reported that allopurinol treatment in isoprenaline-administered rats prevented the LPO and AOPP development in plasma and heart [65]. This investigation further established that the allopurinol and febuxostat treatment in 2K1C rats showed protection against LPO and AOPP development.

NO is often regarded as another oxidative stress mediator. In the presence of superoxide free radicals, NO can transform into peroxynitrite, which is far more hazardous than superoxide itself and causes greater cellular damage. However, NO is crucial for the control of blood pressure, and reduced NO bioactivity is a key factor in hypertension [66]. In our research, we also discovered that allopurinol and febuxostat treatment resulted in a normalization of the elevated NO level in the plasma of 2K1C rats. According to earlier research, 2K1C rats showed higher expression levels of inducible nitric oxide (iNOS) and endothelial nitric oxide synthase (eNOS) [67]. Additionally, prior research has demonstrated that iNOS isoforms have the ability to produce superoxide anions without the need for NO synthesis [68]. The increased NO level in 2K1C rats in this study may be attributed to the increased expression of iNOS isoform in the kidney tissues. This investigation showed that allopurinol and febuxostat treatment normalized the NO level and prevented the iNOS expression in the kidney cortex of 2K1C rats. This investigation is also supported by previous reports showing that allopurinol treatment may alleviate the increased NO level in isoprenaline-administered rats [65].

By examining the markers of oxidative stress, we researched on the protective mechanisms of allopurinol and febuxostat in 2K1C rats. The increased oxidative stress could be a result of declined antioxidant enzymes in the plasma and kidney tissues of 2K1C rats. SOD and CAT activities were found decreased in 2K1C rats. As a result, kidney dysfunction in this model was confirmed by the elevated level of creatinine and uric acid concentration in plasma. In addition, compared to control rats, allopurinol and febuxostat therapy alone significantly increased the antioxidant enzymes like SOD and CAT activities. Furthermore, allopurinol and febuxostat therapy restored the GSH level and reduced LPO. This result is similar to the previous studies showed that XO inhibition may be beneficial in the prevention of kidney complications by improving antioxidant enzymes such as SOD and CAT activities [69–71]. These results lend credence to the idea that allopurinol and febuxostat can shield against oxidative stress sequences.

Increased antioxidant activities in kidneys of 2K1C rats may be due to allopurinol and febuxostat treatment and are direct consequence of restoration of corresponding gene expressions such as SOD, CAT, and GPx. Oxidative stress may jeopardize the gene expression and enzyme level which are involved in scavenging the ROS [53]. Previous report showed that these gene expressions were declined in the kidney cortex in experimental animals [27, 39]. SOD, CAT, and GPx genes were also regulated by Nrf-2 which is a key gene influencing the metabolism and oxidative stress, worked through the expression of other genes like HO-1 and HO-2 [72, 73]. XO inhibitors, in this study, restored the Nrf-2-HO-1 mRNA expression followed by the restoration of SOD, CAT, and GPx gene mRNA expression in the kidney cortex of 2K1C rats. These findings are in line with other reports, suggesting that allopurinol treatment increased the Nrf-2 expression and restored antioxidant defense in diabetic rats [74].

Reactive free radical species–mediated oxidative stress may lead to the infiltration of inflammatory cells and increase the mRNA expression of inflammatory genes such as TNF-α, IL-6, NF-кB, and TGF-β, which are all participate in the progression of kidney inflammation in 2K1C rats. Additionally, in kidneys of 2K1C rats, fibroblast cells and other growth mediators like TGF-β are activated due to inflammation, which may increase the chances of cardiac and renal cell death and initiate fibrosis [36, 75]. This research revealed that kidney tissues have significant deposits of extracellular matrix protein mainly collagen. These results are supported by a previous study suggesting that allopurinol treatment may lower the immune cell infiltration and decreased collagen deposition in experimental animal [19]. Cytokine expression mainly TNF-α and TGF-β signal may activate the fibroblast-like cells which increase the deposition of collagen protein in the kidney [76, 77]. The similar phenomenon was observed in the heart as well, which showed that fibroblast activation due to TNF-α and TGF-β signals may lead to the deposition of collagen in the heart [78]. This collagen deposition could be a reason of increased wet weight of heart and nonclipped kidneys in 2K1C rat. Allopurinol and febuxostat treatment slowed down the inflammation by inhibiting the rise of TNF-α, IL-6, NF-кB, and TGF-β mRNA expression and lowered the production of extracellular matrix protein collagen in the kidneys of 2K1C rats. This finding is also supported by a previous report showing that febuxostat treatment may ameliorate the cytokine production [79] and alleviate the arsenic trioxide–induced renal injury in rats probably by lessening the TNF-α and TGF-β [80].

Molecular docking research is essential for generating a novel medication in structural molecular biology and computer-assisted drug design (CADD), and a molecular docking tool analyzes the prediction of new compound-binding interactions against vital proteins [83]. The molecular docking result showed the binding affinity of the allopurinol and febuxostat with XO; both ligands bind with XO by forming conventional hydrogen bond and Pi-alkyl bonds. As allopurinol and febuxostat are known XO inhibitors, it is predicted that they bound in the inhibitory region of XO. Molecular docking provides the visual representation of the inhibition of XO by allopurinol and febuxostat.

In summary, the allopurinol and febuxostat treatment contributed to the healing process in oxidative stress and inflammation-mediated kidney dysfunction in 2K1C rats. This beneficial effect is dependent on the restoration of antioxidant enzymes through Nrf-2-HO-1-mediated transcriptional regulations in the kidneys. Further research is warranted for deciphering other molecular pathways involved with allopurinol and febuxostat treatment.

## Figures and Tables

**Table 1:** The forward and reverse sequence of the primers that were applied in this experiment [36].

Name of gene	Type	Sequence
Nrf-2	Forward	5′-CCC AGCACA TCC AGACAGAC-3′
	Reverse	5′-TATCCAGGGCAAGCGACT C-3′

Heme oxygenase-1 (HO-1)	Forward	5′-TGCTCGCATGAACACTCTG-3′
	Reverse	5′-TCCTCTGTCAGCAGTGCCT-3′

Heme oxygenase-2 (HO-2)	Forward	5′-CACCACTGCACTTTACTTCA-3′
	Reverse	5′-AGTGCTGGGGAGTTTTAGTG-3′

MnSOD	Forward	5′-GCTCTAATCACGACCCACT-3′
	Reverse	5′-CATTCTCCCAGTTGATTACATTC-3

Catalase	Forward	5′-ATTGCCGTCCGATTCTCC-3′
	Reverse	5′-CCAGTTACCATCTTCAGTGTAG-3′

Glutathione peroxidase (GPx)	Forward	5′-GGGCAAAGAAGATTCCAGGTT-3′
	Reverse	5′-GGACGGCTTCATCTTCAGTGA-3′

IL-1	Forward	5′-ATGCCTCGTGCTGTCTGACC-3′
	Reverse	5′-CCATCTTTAGGAAGACACGGGTT-3′

IL-6	Forward	5′-AGCGATGATGCACTGTCAGA-3′
	Reverse	5′-GGTTTGCCGAGTAGACCTCA-3′

TNF-α	Forward	5′-ATGTGGAACTGGCAGAGGAG-3′
	Reverse	5′-CCACGAGCAGGAATGAGAAGAG-3′

TGF-β	Forward	5′-AAGAAGTCACCCGCGTGCTA-3′
	Reverse	5′-TGTGTGATGTCTTTGGTTTTGTC-3′

iNOS	Forward	5′-TGGTCCAACCTGCAGGTCTTC-3′
	Reverse	5′-CAGTAATGGCCGACCTGATGTTG-3′

NF-кB	Forward	5′-TGTGAAGAAGCGAGACCTGGAG-3′
	Reverse	5′-GGCACGGTTATCAAAAATCGGATG-3′

β-actin	Forward	5′-GCGAGAAGATGACCCAGATC-3′
	Reverse	5′-GGATAGCACAGCCTGGATAG-3′

**Table 2:** Binding parameters for allopurinol and febuxostat with human milk oxidoreductase/xanthine oxidase proteins in molecular docking analysis.

Receptor	Ligand	Binding affinity	Bond type	Amino acid residue	Distance (Å)
Human milk xanthine oxidoreductase/xanthine oxidase (PDB ID: 2CKJ)	Allopurinol (PubChem ID: 135401907)	−5.9 kcal/mol	Conventional hydrogen bond	GLY C: 260	2.29
				ASN C: 261	2.05
				THR C: 354	2.01
			Pi-alkyl bond	VAL C: 259	5.32
				ALA C: 346	4.94
	Febuxostat (PubChem ID: 134018)	−8.00 kcal/mol	Conventional hydrogen bond	LYS C: 256	4.2
				ASN C: 261	2.06
				SER C: 347	2.66
			Pi-alkyl bond	LEU C: 257	4.33
				VAL C: 259	5.47
				ILE C: 353	4.47
			Alkyl bond	LEU C: 398	3.93

## Data Availability

The data are available within the manuscript. Further information can be obtained from the corresponding author upon reasonable request.
